# svBreak: A New Approach for the Detection of Structural Variant Breakpoints Based on Convolutional Neural Network

**DOI:** 10.1155/2022/7196040

**Published:** 2022-03-19

**Authors:** Shaoqiang Wang, Jie Li, A K Alvi Haque, Haiyong Zhao, Liying Yang, Xiguo Yuan

**Affiliations:** ^1^School of Computer Science and Technology, Xidian University, Taibai South Road, Xi'an, Shannxi Province, China; ^2^Hangzhou Institute of Technology, Xidian University, Hangzhou, China; ^3^Business School, Xi'an Fanyi University, Changan District Taiyi Palace, Xi'an, Shannxi Province, China; ^4^School of Computer Science and Technology, Liaocheng University, Liaocheng, Shandong Province, China

## Abstract

Structural variation (SV) is an important type of genome variation and confers susceptibility to human cancer diseases. Systematic analysis of SVs has become a crucial step for the exploration of mechanisms and precision diagnosis of cancers. The central point is how to accurately detect SV breakpoints by using next-generation sequencing (NGS) data. Due to the cooccurrence of multiple types of SVs in the human genome and the intrinsic complexity of SVs, the discrimination of SV breakpoint types is a challenging task. In this paper, we propose a convolutional neural network- (CNN-) based approach, called svBreak, for the detection and discrimination of common types of SV breakpoints. The principle of svBreak is that it extracts a set of SV-related features for each genome site from the sequencing reads aligned to the reference genome and establishes a data matrix where each row represents one site and each column represents one feature and then adopts a CNN model to analyze such data matrix for the prediction of SV breakpoints. The performance of the proposed approach is tested via simulation studies and application to a real sequencing sample. The experimental results demonstrate the merits of the proposed approach when compared with existing methods. Thus, svBreak can be expected to be a supplementary approach in the field of SV analysis in human tumor genomes.

## 1. Introduction

**Figure 1:**
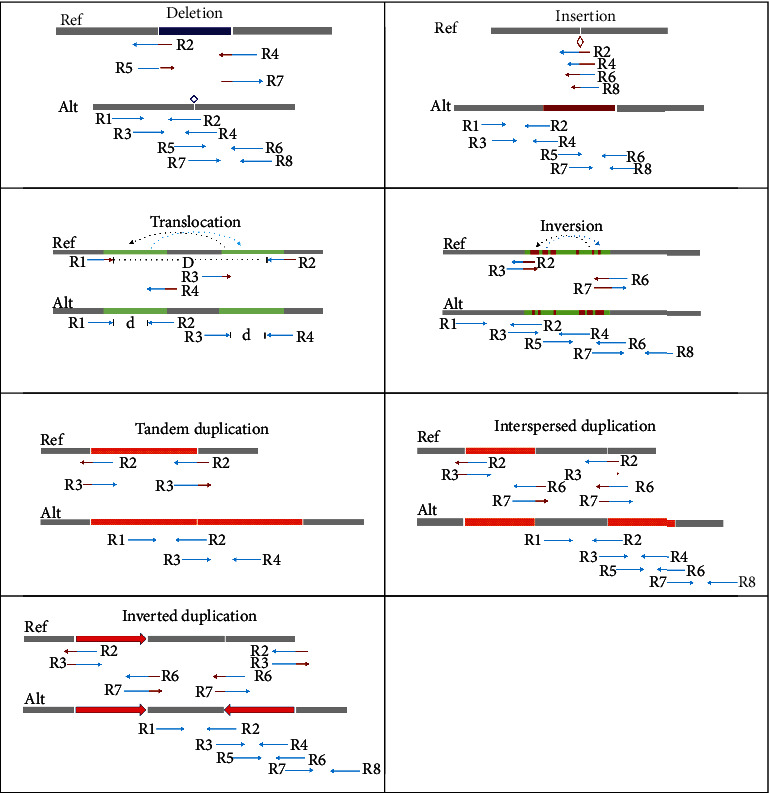
Seven common categories of structural variations.

Structural variations (SVs) are very common in human genome, and their sizes are ranging in a large interval from several base pairs (bps) to ten thousand bps or even more. Accurate detection of SVs could provide variation information for the exploration of mechanisms and precision diagnosis of cancers [1–3]. Generally, SVs can be classified into various categories according to their characteristics. In Figure 1, we describe seven common categories of SVs by observing the statuses of sequencing reads aligned to the human reference genome. These SVs include insertion, deletion, translocation, inversion, interspersed duplication, inverted duplication, and tandem duplication [4]. Discrimination of these SV categories in the human genome would be necessary and meaningful for a deep analysis of the landscape of genome mutations. Moreover, the inference of clonal and subclonal structures has a significant influence on cancer research [5, 6]. Along with the extremely high resolution (at base pair-level resolution) data provided by next-generation sequencing (NGS) technologies, the detection of precision breakpoints of these SVs has become feasible.

Currently, a number of computational and statistical methods have been developed for the detection of SVs. By using the information of short reads from NGS data, these methods could be roughly categorized into five different categories: paired-end mapping (PEM), split-read (SR), read depth (RD), de novo assembly (DA), and hybrid approaches [7, 8]. The basic idea of PEM-based methods is to determine the size and location of SVs by comparing the mapping distance (distance between the paired reads) with insert size. Classic PEM-based methods include Delly [9] and Breakdancer [10]. These methods have the advantage of detecting SVs of short lengths with high accuracy. However, the exact locations (base pair level) of SVs are difficult to detect using such methods. The basic idea of the SR-based methods is to use the splitting points (between matched and unmatched parts) of reads to determine SV breakpoints and to use the mapping positions of the reads to deduce SV contents. Traditional SR-based methods include Pindel [11] and SVseq2 [12]. The most enticing advantage of these methods is that they can precisely detect breakpoints of SVs. Although these methods usually perform well in detecting short SVs, their performances in detecting large SVs are limited. RD-based methods use read count fluctuation information across the genome to determine the contents and positions of SVs. Classic RD-based methods include FREEC [13], CONDEL [14], CNV_IFTV [15], WaveDec [16], and iCopyDAV [17]. These methods have the ability to detect large sizes of SVs but cannot precisely detect SV breakpoints. The main idea of the DA-based methods is to generate large contigs by assembling short reads and then mapping the contigs to the reference genome for inferring SVs. Traditional DA-based methods include IMSindel [18] and SOAPindel [19]. These methods can theoretically detect many types of SVs with a large range of sizes, but the assembly of contigs can be affected by multiple factors, such as sequencing depth, mapping errors, and heavy computing resources. The hybrid-based approach is a combination of two or more categories of the aforementioned methods described above. Classic methods of such category include SoftSV [20] and Scalpel [21]. The advantage of such an approach is that it can integrate different types of mapped reads to improve the sensitivity of SV detection. Nevertheless, the use of multiple types of information means that there will be more SV candidates to be discriminated against. On the whole, the existing methods have their advantages in the detection of SVs from tumor samples, but few of them have been designed to detect most of the common SVs simultaneously.

With careful consideration of the issues described above, we propose an alternative approach, called svBreak, for the detection of SV breakpoints from NGS data. This approach is established based on a convolutional neural network (CNN) [22]. The core principle of svBreak is that it extracts a set of SV-related features for each genome site from the sequencing reads aligned to the reference genome and creates a data matrix where each row represents one site and each column represents one feature and then adopts a CNN model to analyze such data matrix for the prediction of SV breakpoints. The svBreak method can simultaneously detect and discriminate breakpoints of the seven common types of SVs.

## 2. Materials and Methods

### 2.1. Flowchart of svBreak

**Figure 2:**
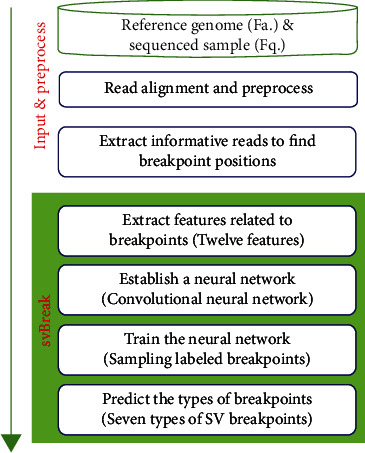
The flowchart of svBreak.

The overall workflow of the svBreak method is illustrated in Figure 2. It starts with an initial input of a reference genome and a sequencing sample and then adopts a classic tool such as BWA [23] to perform the alignment process. The reference genome can be chosen from official references, such as human genome (HG19) or the latest version, and the sequencing sample to be analyzed can be obtained from synthetic or real NGS data. Based on the alignment result, the reads with low mapping quality are filtered and informative reads are extracted for discovering breakpoint positions. Subsequently, svBreak carries out four primary steps to predict SV breakpoints. The four primary steps include (1) extracting twelve distinctive features related to SV breakpoints, (2) establishing a CNN model based on the topology of the AlexNet model [24] for the analysis of the extracted features, (3) training the neural network by using labeled SV breakpoints from synthetic or real NGS data, and (4) predicting the seven common types of SV breakpoints based on the trained CNN model. In the following text, we make a detailed description of the principle for each of the steps.

### 2.2. Input and Preprocessing

Before describing the principles of the steps above, we make a brief introduction to the preprocessing of the input data. Currently, the main human reference genome databases include the NCBI database, UCSC Genome Database, and Ensembl Genome Database. The human reference genome of the UCSC Genome Database mainly includes HG18, HG19, and HG38 versions. Since HG19 can provide more gene annotation information and this version of the human reference genome has been widely used by most existing research institutes, we adopt the HG19 as the reference genome for the alignment of the NGS data. The aligned data is then filtered by low-quality reads, and the high-quality and informative reads including split reads and discordant reads are extracted which generates a SAM file for the input to the core module of svBreak.

### 2.3. Extracting Features Related to SV Breakpoints

Based on the above split and discordant reads of the SAM file, we extract twelve features related to SV breakpoints, which are shown in Table 1. Each of the features can be assigned with the values of 1, 0, or -1 according to the status on each genome site. The value of 1 means that the status of the feature is positive, -1 means a negative status of the feature, and 0 means an uncertainty status. For example, for the first feature, the existence of reversely mapped reads (RMR) means whether there exist some reads that are reversely mapped on one site. If yes, the value of the feature RMR is 1, else, it is 0. The uncertainty status of one feature means that the specific feature cannot be determined by the currently aligned reads. For example, we did not find any reversely mapped reads in one site. This might be attributed to a lack of reads due to limited sequencing coverage depth. In this case, the value of the feature RMR in this site was set to 0. The value of each feature is explained in Table 1. Moreover, the concepts of MS and SM are explained below. For example, for a read with a length of 100 bp, if its alignment result displays “70M30S,” it means that the first segment of the result with 70 bp matched with the reference genome and the other part of the result with 30 bp is soft mapped (*i.e.*, not matched). Such a type of alignment is called MS. If the alignment result displays “30S70M,” then such alignment is called SM.

### 2.4. Establishing a Convolutional Neural Network

With the aforementioned extracted feature, we construct a CNN model for the prediction of SV breakpoints. Specifically, for each type of SV breakpoint (*e.g.*, insertion), we trained a CNN model for the classification on each genome site. Thus, for the seven common types of SV breakpoints, we train a total of seven CNN models, each of which is a binary classification model. Here, the CNN classification model generates a score for each genome site. For example, for a genome site *t*, the insertion-specific CNN model will generate a score *s*_1_^*t*^. The larger the score is, the higher the probability that the genome site belongs to an insertion breakpoint. Meanwhile, the other six SV type-specific CNN models also generate scores *s*_*i*_^*t*^ (*i* = 2, 3, ⋯, 7) for genome site *t*. Finally, determining which type of SV breakpoint the genome site *t* belongs to depends on the largest score among the seven scores. If *s*_1_^*t*^ displays the largest score, then the genome site *t* belongs to the insertion breakpoint. Nevertheless, if all the seven scores display zeros, then the genome site *t* is not an SV breakpoint.

**Figure 3:**
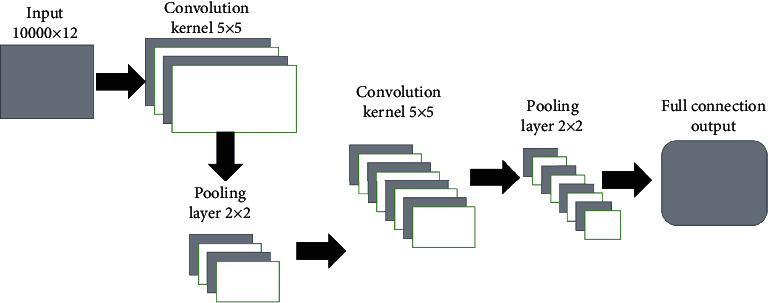
The topology of convolutional neural network.

For a clear understanding of the CNN model, we depict the topology of the CNN model in Figure 3. The CNN model is a multilayer perceptron with a deep learning model. The input data to the CNN model is a two-dimensional data matrix, *M*_10000×12_, where each row represents one genome site and each column represents one feature. Such input data matrix means that the prediction of SV breakpoints is carried out batch by batch, each of which contains 10000 sites in the genome. Before training the CNN model, the data matrix in the input layer is preprocessed via the following two steps: (1) batch normalization [19], centralizing the values on each column to zero, which aim to pull the center of the sample back to the origin of the coordinate system and (2) scaling the values from different columns to the same range, which can help to make a balanced trade-off between different feature values.

The CNN model in Figure 3 is further described below. The convolutional layer uses the convolution kernel for feature extraction and feature mapping, which is the most important layer in the CNN model. At this layer, two key operations are carried out: (1) localizing correlation and sliding window and (2) using convolution to check local data calculation. Here, the size of the convolution kernel is set to 5 × 5. We use the Gaussian initial and set the step size to be 1. The excitation layer will perform a weighted nonlinear mapping on the output matrix. Since the excitation layer has a single function and needs to work with the convolutional layer to control parameters, the excitation layer is often hidden in the convolutional layer in the structure diagram. The purpose of this layer is to iteratively train the neural network and adjust the parameters according to the results of the negative feedback of the neural network. The excitation layer needs to use activation functions to adjust parameters. We choose the ReLU function [20] as the activation function rather than the Sigmoid function, which may cause the gradient to disappear. The pooling layer is to conduct downsampling, sparse processing of feature maps, and reducing the amount of data computation. We set the size of the pooling layer to 2 × 2. In the output layer, we adopt a full connection, which can reduce the loss of feature information. In a fully connected layer, many-to-many connections between neurons are formed, that is, neurons in each layer are connected to all neurons in other layers, and these connections are all right edges. The number of layers of a convolutional neural network is generally defined as the sum of levels with parameters. The neural network established in the algorithm of this paper contains five layers of convolutional layers and three layers of fully connected layers.

### 2.5. Training the CNN Model

As far as the training of the CNN model is concerned, we use a total of 50,000 groups of samples, each of which contains all the seven types of SV breakpoints. Each genome site of the samples is labeled as some type of SV breakpoints or normal status (*i.e.*, nonbreakpoint genome site). In the experiment, in order to reduce overfitting, we carry out cyclic cross-validation at a ratio of 8 : 2, *i.e.*, eighty percent of the samples were used for training, and twenty percent of the samples were used for testing. Theoretically, the training samples could be obtained from either synthetic or real sequencing individuals. However, in real sequencing individuals, the SV breakpoints are usually difficult to obtain. Thus, in our experiments, we choose to make a simulation of SV breakpoints for the training of the CNN model.

Since there are seven common types of structural variation, this paper trained seven classifiers of convolutional neural network models. When training a certain convolutional neural network model, the SV breakpoints corresponding to the model are regarded as positive samples, while the remaining SV breakpoints and noise sites are regarded as negative samples. Each classifier model will give a score value. The larger the score, the higher the probability that the breakpoint belongs to the structural variation represented by the classifier, and we get a total of seven such scores. Finally, we choose the highest score among the seven scores obtained, and the breakpoint is divided into the SV type corresponding to the highest score classifier.

## 3. Results

The svBreak software is implemented in both Python and Java languages under the Linux system. The source code and manual of the svBreak software package are publicly available at https://github.com/BDanalysis/svBreak. It is very important to adopt a reasonable way to assess the performance of svBreak. Simulation studies are considered a feasible approach for this task [25, 26] since simulation can provide ground truth SVs for the quantification of sensitivity and precision of the method. Here, we perform simulation studies for the svBreak method and make a comparison with two currently popular methods Tardis [27] and TIDDIT [28] with respect to their precision and sensitivity. Furthermore, we apply the svBreak method to real sequencing data to validate its usefulness.

### 3.1. Simulation Studies

**Figure 4:**
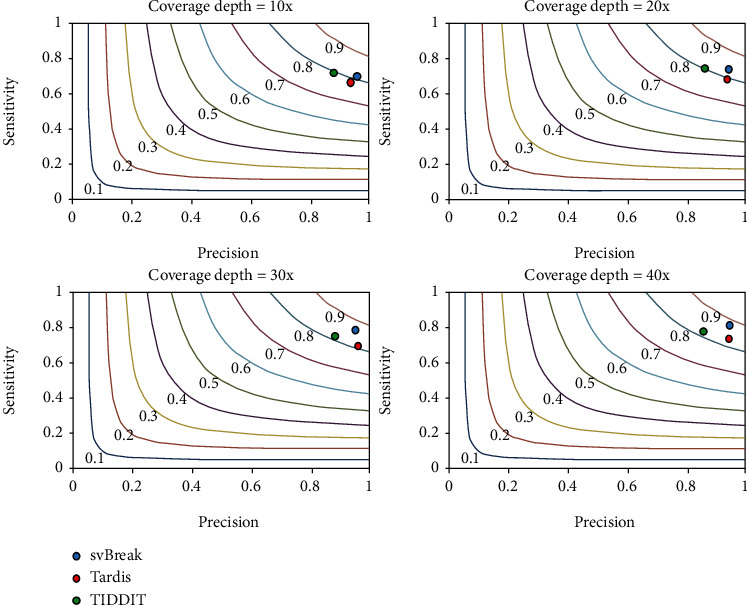
Performance comparison between the three methods in terms of sensitivity, precision, and *F*1-score on simulation datasets.

We use one of the classic and popular simulation software SInC [29] to generate SVs and sequencing reads. To generate a variety of simulation data, we set the coverage depth to 10x, 20x, 30x, and 40x, respectively. In each configuration of coverage depth, we generate fifty replications for testing the svBreak method and the two compared methods. The comparative results are presented in Figure 4. Here, the sensitivity is calculated as the ratio of the number of correctly predicted SV breakpoints to the total number of simulated SV breakpoints, and the precision is calculated as the ratio of the number of correctly predicted SV breakpoints to the total number of predictions. The *F*1-score (colored curves) is the harmonic mean of sensitivity and precision. Here, the presented sensitivity and precision are the averaged values over the fifty replications in each simulation configuration. The comparative results indicate that the performances (sensitivity, precision, and *F*1-score) of the three methods are improving when the coverage depth is increasing. For instance, the *F*1-score of the Tardis method is below 0.9 when the coverage depth is 10x while the value is over 0.9 when the coverage depth is 40x. Among the three methods, svBreak has achieved a relatively higher *F*1-score than the other two methods under each configuration of the coverage depths. In terms of sensitivity, TIDDIT performs best when the coverage depths are 10x and 20x while svBreak performs best when the coverage depths are 30x and 40x. In terms of precision, svBreak and Tardis are superior in all four configurations of coverage depths. On the whole, svBreak has obtained the best trade-off between sensitivity and precision in these simulation experiments.

**Figure 5:**
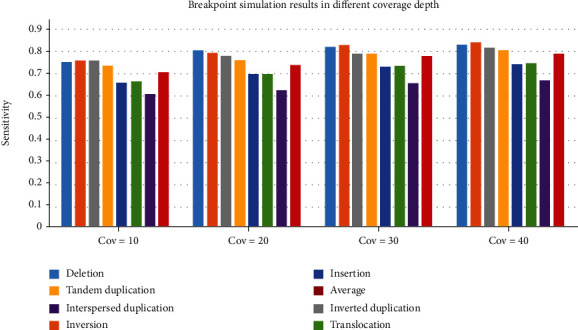
Seven types of structural variation sensitivity on simulation data of coverage from 10x to 40x.

Moreover, considering that the seven common types of SV breakpoints may have different effects, it is meaningful to explore the performance of the svBreak method in detecting each type of the SV breakpoints. For this, we calculate the sensitivity for the detection of each type of the SV breakpoints in the experiments. The result is shown in Figure 5. It can be observed that, in each configuration of the coverage depths, the breakpoints of deletion, inversion, inverted duplication, and tandem duplication are detected at larger sensitivities than the other three types of SV breakpoints, while the breakpoint of interspersed duplication is detected at the lowest sensitivity. The sensitivity of the detection of interspersed duplication, insertion, and translocation is relatively low, but as the sequencing depth increases, the detection of these types of mutations has a significant improvement. When the sequencing depth had been increased from 10x to 40x, the sensitivity of detecting the breakpoint of interspersed duplication, insertion, and translocation have increased by 10.6%, 12.9%, and 12.6%, respectively, which is larger than the other four mutation types. The physical structure of these three variant types is relatively complex. Under the condition of low sequencing depth, the detection effect is poor, but with the increase of sequencing depth, the improvement of the detection effect is more obvious. The increase in sensitivity of the detection of insertion and translocation mutations exceeds the average increase in sensitivity by more than 2%. This means that these two structural variations may be greatly affected by the sequencing depth. Note that more statistical testing on the simulation dataset is shown in the supplementary material (available here) where the impact of coverage on sensitivity is observed.

The reason for the high accuracy of the svBreak algorithm in this paper is that its feature values are mainly obtained from the comparison information of split reads and the insertion distance of paired-end sequencing reads. The use of comparison information to judge means that a screening has already been carried out before the classification process. Therefore, part of the wrong breakpoint information is filtered out. Moreover, this paper takes the “existence of reverse alignment” as a feature, so it becomes easier to distinguish between inverted mutations and inverted repeat mutations that exist in reverse aligned reads. The sensitivity of deletion mutations is relatively high because split reads are very sensitive to deletions. This article also extracts features related to split reads, so it is more sensitive to deletion mutations.

### 3.2. Real Data Application

**Figure 6:**
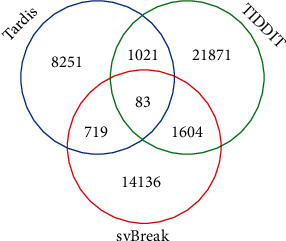
The overlapping result among the three methods.

To validate the usefulness of the svBreak method, we apply it to analyze a real sequencing sample with ID No. EGAD00001000144_LC. This sample was obtained from the European Genome-phenome Archive and sequenced from a lung cancer patient. For comparison, we carry out the Tardis and TIDDIT methods on this sample. The comparative result is depicted in Figure 6. From this figure, we can notice that TIDDIT gets the largest number of calls while Tardis gets the smallest number of calls. Generally, it will be not reasonable to judge the performance of the methods only according to the number of calls. Since we usually do not know the ground truth SV breakpoints in real samples, it is difficult to calculate the sensitivity or precision of the methods. Instead, we use our previously proposed overlapping density score (ODS) [14] to quantify the methods. ODS can be used to analyze the consistency of the results among multiple methods. The higher the ODS value of a method, the better its performance. The formula for calculating ODS values can be referred to [14]. After calculation, the obtained values of ODS for the svBreak, Tardis, and TIDDIT methods are 190.9, 183.5, and 99.4, respectively. This means that svBreak can achieve a greater consistent result than the other two methods. Thus, we may conclude that svBreak is a useful method in the analysis of SVs in the human genome.

## 4. Conclusion

In this paper, we propose an alternative method called svBreak for the prediction of SV breakpoints in the human genome. The application of deep learning models has made notable breakthroughs in bioinformatics modeling [30, 31]. This paper combines convolutional neural networks with bioinformatics and uses convolutional neural networks to classify SV breakpoints. The central characteristic of our proposed method is that it extracts twelve SV-related features for each genome site from the sequencing reads aligned to the reference genome and adopts a CNN model for SV breakpoint prediction. In order to further improve the performance of the convolutional neural network, this paper adds a large number of labels to the training set. With this part of the prior knowledge, the neural network will be more sensitive to this kind of data. Another reason for choosing convolutional neural networks is that convolutional neural networks have fewer learnable parameters than standard fully connected neural networks in structure, so convolutional neural networks are easier to train and less subject to overfitting. svBreak is able to detect and discriminate seven common SV breakpoints and is tested using simulation and real sequencing data. The experimental results demonstrate that svBreak is a valid and useful method. Thus, svBreak can be expected to be a supplementary approach in the field of SV analysis in human genomes.

In the future work, we intend to extend the current version of svBreak from the following three perspectives. In the first place, the detection of SV breakpoints can be influenced by the contamination of normal genomes in tumor genomes to be analyzed; thus, estimating tumor purity and recovering tumor genome signals will facilitate the detection of SV breakpoints. In the second place, the detection of SV breakpoints should be combined with the detection of single nucleotide variations [32] for the improvement of genomic mutations. In the last place, the concept of semisupervised learning could be introduced into the training of svBreak, to improve the generalization performance of svBreak.

## Figures and Tables

**Table 1:** Description of the extracted twelve features.

Features	Description
Reversely mapped read (RMR)	If such read exists on one genome site, the value of this feature on the site is 1; otherwise, it is 0
Reversely mapped read showing SM (RMSM)	If such read exists on one genome site, the value of this feature on the site is 1; otherwise, it is -1
Reversely mapped read showing MS (RMMS)	If such read exists on one genome site, the value of this feature on the site is 1; otherwise, it is -1
Mapped read showing SM (MSM)	If such read exists on one genome site, the value of this feature on the site is 1; otherwise, it is 0
Mapped read showing MS (MMS)	If such read exists on one genome site, the value of this feature on the site is 1; otherwise, it is 0
The mapped distance between the paired-end reads smaller than insert size (MDS)	If such paired-end reads exist on one genome site, the value of this feature on the site is 1; otherwise, it is -1
The mapped distance between the paired-end reads equal to insert size (MDE)	If such paired-end reads exist g on one genome site, the value of this feature on the site is 1; otherwise, it is -1
The mapped distance between the paired-end reads larger than insert size (MDL)	If such paired-end reads exist on one genome site, the value of this feature on the site is 1; otherwise, it is -1
Previous breakpoint site with mapped reads showing SM (PSM)	If such read exists on one genome site, the value of this feature on the site is 1; otherwise, it is 0
Previous breakpoint site with mapped reads showing MS (PMS)	If such read exists on one genome site, the value of this feature on the site is 1; otherwise, it is 0
Next breakpoint site with mapped reads showing SM (NSM)	If such read exists on one genome site, the value of this feature on the site is 1; otherwise, it is 0
Next breakpoint site with mapped reads showing MS (NMS)	If such read exists on one genome site, the value of this feature on the site is 1; otherwise, it is 0

## Data Availability

Data is available at https://ega-archive.org/.

## Supplementary Materials

Supplementary Materials Statistical experiment on simulation dataset. Figure 1: multiple experiments of svBreak algorithm on the simulation dataset.
