# Supplementary material for: svBreak: A New Approach for the Detection of Structural Variant Breakpoints Based on Convolutional Neural Network
Source: Biomed Res Int. 2022 Mar 19;2022:7196040. doi: 10.1155/2022/7196040 (PMC8957449; doi:10.1155/2022/7196040)
Supplement: Supplementary Materials — Statistical experiment on simulation dataset. Figure 1: multiple experiments of svBreak algorithm on the simulation dataset. [file 7196040.f1.docx]

***Supplementary Material***

# **Statistical Experiment on Simulation Dataset**

To further verify the stability of the svBreak method under different sequencing depths, we draw a line graph with the sequencing depth ranging from 10x to 40x, as shown in Figure 1. Here we mainly study the impact of coverage on sensitivity. It can be seen from Figure 1 that when the sequencing depth is low, the sensitivity difference between multiple experimental results is large, which means that at a low sequencing depth, the standard deviation of the svBreak algorithm is large. As the coverage increases, the standard deviation is significantly reduced, and the results of multiple experiments are relatively stable.


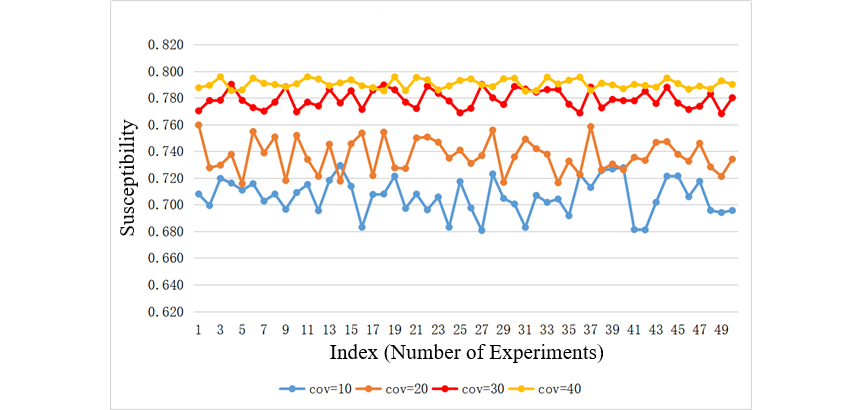


Figure 1 Multiple experiments of svBreak algorithm on simulation dataset

In short, during the simulation experiment, the accuracy of the svBreak algorithm and the Tardis algorithm are relatively stable. Under the different sequencing depths set in this article, the accuracy can reach more than 0.95. The accuracy of the TIDDIT algorithm is slightly lower than the other two algorithms, close to 0.9.
